# Isolation, Characterization, and Comparative Analysis of Two Subtypes of Goose Astrovirus in Guangdong Province, China

**DOI:** 10.3390/microorganisms13051037

**Published:** 2025-04-30

**Authors:** Chenggang Liu, Linlin Li, Jiawen Dong, Jin Jin, Yong Xiang, Junqin Zhang, Qi Zhai, Yunzhen Huang, Binyi Sun, Ming Liao, Minhua Sun

**Affiliations:** 1 Institute of Animal Health, Guangdong Academy of Agricultural Sciences, Key Laboratory for Prevention and Control of Avian Influenza and Other Major Poultry Diseases, Ministry of Agriculture and Rural Affairs, Key Laboratory of Livestock Disease Prevention and Treatment of Guangdong Province, Guangzhou 510640, China; lcg85@126.com (C.L.); lingdang1000@163.com (L.L.); hellendongjiawen@163.com (J.D.); xiangyongcq@163.com (Y.X.); junqinzhang@yeah.net (J.Z.); zhaiqi@gdaas.cn (Q.Z.); huangyunzhen110@hotmail.com (Y.H.); mliao@scau.edu.cn (M.L.); 2 Shanwei Academy of Agricultural Sciences, Shanwei 516699, China; msj.jin@hotmail.com (J.J.); sunbinyi00@126.com (B.S.)

**Keywords:** goose astrovirus, gout, phylogenetic analysis, tissue tropism, protein structure

## Abstract

Since 2017, an infectious disease characterized by gosling gout and caused by goose astrovirus (GAstV) has affected geese in most major goose-producing regions of China. In this study, a total of 385 geese displaying gout symptoms were sampled from 12 cities in Guangdong Province, China, between 2019 and 2021. RT-PCR analysis revealed that all samples were positive for GAstV (385/385), with GAstV-II being the predominant subtype, accounting for 90.4% (348/385) of the cases. Co-infection with GAstV-I and GAstV-II was detected in 50.4% (194/385) of the samples. Additionally, different GAstV subtypes were successfully isolated using goose embryos, namely GDYJ-21-01 (GAstV-I) and GDZJ-21-01 (GAstV-II). Analysis of viral copy numbers in major pathological tissues following infection of goslings and goose embryos revealed that GDZJ strain exhibited broader tissue tropism than GDYJ strain. Compared to other tissues, GDYJ strain displayed tissue tropism exclusively in the cecal tonsils of goslings and the allantoic fluid of embryos. Structural prediction and alignment using AlphaFold 2.0 identified an α-helix in the S223-A226 region of the GDZJ VP34 protein, while a loop structure was observed in the Q235-Q237 region of the corresponding GDYJ VP34 protein. Furthermore, although the VP27 protein regions of both subtypes contained five β-sheet structures, the overall sequence similarity was relatively low, at 37.1%. This study broadens our understanding of the prevalence differences among GAstV subtypes and provides valuable insights into the development of reagents for preventing these viral infections.

## 1. Introduction

Since 2017, the first reported case of gout in goslings caused by goose astrovirus (GAstV) was documented along the southeastern coast of China [1,2,3]. The main characteristic of this disease is the deposition of urate salts in the internal organs and joints of poultry [4,5,6]. The GAstV was quickly spread to inland provinces in China within a short period [7]. Since then, gout in goslings caused by GAstV has become a widespread infectious disease, significantly impeding the development of the goose industry.

GAstV has been identified as a major pathogen associated with gout symptoms in poultry [2,8]. The virus primarily infects goslings under 20 days old [9], with a mortality rate exceeding 50.0% in infected individuals [4,6]. Research has shown that GAstV can cross species barriers, infecting other poultry, including chickens and ducks [10,11]. The GAstV is transmitted not only through fecal–oral routes, but also vertically [12]. The diversity of transmission routes increases the risk in poultry farming, leading to significant economic losses, particularly in the goose industry.

GAstV is a member of the *Astroviridae* family, a non-enveloped, single-stranded positive-sense RNA virus [7]. Its genome consists of a 5′-untranslated region (UTR), three open reading frames (ORFs: ORF1a, ORF1b, and ORF2), a 3′-UTR, and a poly (A) sequence [13]. ORF1a and ORF1b encode non-structural viral proteins [14], while ORF2 encodes the viral capsid protein, which exhibits the greatest diversity within the genome [15]. Based on the molecular characteristics and genetic evolution analysis of ORF2, GAstV is classified into two subtypes: GAstV-I and GAstV-II [15,16]. ORF2 is a key protein involved in various processes, including the assembly of astrovirus viruses [17,18], recognition of cell surface associated receptors [19], host immune response [20], and facilitation of viral invasion [21]. Studies have shown that ORF2 plays a key role in the tissue tropism of the virus [22,23].

Research has shown that GAstV induces gout in goslings by modulating host metabolic reprogramming [24]. Additionally, GAstV infection triggers apoptosis and an inflammatory response in goose embryonic kidney cells [25]. Both GAstV-I and GAstV-II are capable of causing gout in goslings [6,26]. However, a recent study has shown that two subtypes of GAstV elicit distinct response patterns in goose embryonic fibroblasts, with GAstV-I triggering a higher innate immune response in the host, while GAstV-II exerts a more pronounced effect on the host’s metabolic pathways [27]. As such, understanding the differences in infectivity and tissue tropism between these two subtypes of GAstV is critical.

In this study, 385 clinical samples of geese exhibiting gout symptoms were collected between 2019 and 2021 to examine the epidemiological characteristics of GAstV in Guangdong Province. The pathogenicity of the isolated strains was verified through experimental infection studies on both GDYJ (GAstV-I) and GDZJ (GAstV-II). Additionally, the possible reasons for the tissue tropism differences between GAstV-I and GAstV-II were investigated through prediction and analysis of the ORF2 protein structure. Finally, we hope these results can provide a solid foundation for future exploration of the pathogenic mechanisms of GAstV.

## 2. Materials and Methods

### 2.1. Sample Collection, Nucleic Acid Extraction, and GAstV Identification

From 2019 to 2021, a total of 385 samples were collected from geese exhibiting symptoms of gout across 44 farms in Guangdong Province. Samples were obtained from major goose breeds, such as Magang geese, lion-headed geese, black mane geese, and Baisha geese. Tissue samples were stored at −80 °C until analysis.

For each sample, 0.1 g of tissue (liver or kidney) was homogenized with 1 mL of sterile saline using a tissue-grinding homogenizer (TIANGEN, Beijing, China). The mixture was then centrifuged for 10 min at 12,000× *g* and 4 °C, and the supernatant was collected. The supernatant was used for nucleic acid extraction and viral culture. Viral nucleic acids were extracted from tissue supernatants using an automatic nucleic acid extraction instrument and its accompanying extraction kit (TIANLONG, Xi’an, China). The number of infections of GAstVs, goose parvovirus (GPV), goose hemorrhagic polyomavirus (GHPV), goose tembusu virus (TMUV), avian adenovirus (FAdV), goose reovirus (GRV), and goose circovirus (GoCV) in the samples were determined using reverse transcription polymerase chain reaction (RT-PCR) or PCR with the identification primers described by Wang et al. [28,29]. AceTaq DNA Polymerase (Vazyme, Nanjing, China) is used to amplify DNA viral nucleic acids, while HiScript II One Step RT-PCR Kit (Dye Plus) (Vazyme, Nanjing, China) is used to amplify RNA viral nucleic acids. The reaction system and procedure are carried out according to the manufacturer’s instructions. The full-length ORF2 genes from different genotypes of GAstVs were identified by RT-PCR using the primers listed in Table 1.

### 2.2. Clinical and Pathological Features, and Hematoxylin and Eosin (H and E) Staining

Necropsy was performed on clinically diseased goslings to observe tissues susceptible to urate deposition (e.g., heart, joints, gallbladder) and other lesions. Tissue samples, including kidney, liver, spleen, heart, small intestine, cecum, and colon, were collected for histopathological analysis. Tissue samples from age-matched gosling without symptoms of gout and negative for GAstVs detection were used as negative controls. Samples were fixed in 4.0% paraformaldehyde at room temperature and subsequently sent to Wuhan Servicebio Technology Co., Ltd. (Wuhan, China) for paraffin section preparation, H and E staining, and panoramic section scanning. CaseViewer software (CaseViewer 2.4, 3DHISTECH Ltd., Budapest, Hungary) was used to analyze the scan results and perform histopathological evaluations of the stained sections.

### 2.3. Isolation of GAstVs

The supernatants from tissue samples infected solely with GAstV-I or GAstV-II were filtered through a 0.45 μm filter, and 300 μL of the filtrate was inoculated onto a chorioallantoic membrane of 11-day-old goose embryos, which were then sealed with sterile medical tape. Embryos were then incubated at 37 °C. After 5 days post-inoculation (dpi), nucleic acids were extracted from the allantoic fluid, and GAstV was detected using RT-PCR. Subsequently, 300 μL of allantoic fluid from GAstV-positive embryos was re-inoculated into 11-day-old goose embryos, with this process being repeated for a total of five passages.

### 2.4. Goose Embryos and Goslings

The 10-day-old goose embryos and 1-day-old goslings utilized in this study were procured from a goose breeding farm located near Guangzhou, Guangdong Province. Prior to the experiment, embryos and goslings were confirmed to be negative for common gosling infection viruses, including GAstVs. Briefly, for 1-day-old goslings, collect throat and cloacal swabs using sterile swabs, dissolve them in 1 mL sterile PBS, shake well, and take 200 μL for the next step of nucleic acid extraction. For 10-day-old goose embryos, 200 μL of allantoic fluid is aspirated sterilely before infection. Viral nucleic acid is extracted from 200 μL of throat and cloacal swab or allantoic fluid using an automatic nucleic acid extraction instrument and its accompanying extraction kit (TIANLONG, Xi’an, China). The types and methods of virus detection are detailed in chapter 2.1. Goslings and goose embryos that tested negative for common viruses were used in the subsequent experiments.

### 2.5. Genome Sequencing and Phylogenetic Analysis

The primers and methodologies used to amplify the complete genomes of GAstV isolates were described previously [15]. The evolutionary relationships of the complete genomic sequences of different astrovirus species were analyzed using the Neighbor-Joining method in Molecular Evolutionary Genetics Analysis (MEGA, version 11.0) software [30]. The complete genome sequences of the isolated GAstV strains have been deposited in the GenBank database under the accession numbers OP776630.1 (GDYJ-21-01) and OP776629.1 (GDZJ-21-01).

### 2.6. Recombination Analysis

Retrieve reference sequences of GAstVs from the GenBank database. The phylogenetic tree based on the full-length genome sequence was constructed using MEGA 11.0 software with 1000 bootstrap replicates. Recombination analysis and calculation were performed using RDP (4.101) software with multiple algorithms, including RDP, Bootscan, and GENECONV. The RDP step size was set to 30, with a *p*-value threshold of <0.05, and the Bonferroni method was applied for correction. For sliding window analysis, SimPlot+Windows V1.3 software (JHK University, Baltimore, MD, USA) was used, with a step size of 50 and a window length of 250.

### 2.7. Infection Experiment

Thirty 3-day-old healthy goslings were divided into three groups (10 goslings per group). Goslings in the first group each received an intramuscular injection (0.2 mL suspension containing a virus load equal to 5.0 × 10^9^ copies) of 5th generation GDYJ-21-01 (GAstV-I); those in the second group were intramuscularly injected (0.2 mL suspension containing a virus load equal to 5.0 × 10^9^ copies) with GDZJ-21-01 (GAstV-II); and those in the third group received 0.2 mL of sterile saline. Each group was housed in a separate room, and goslings had free access to food and water. Throat and cloacal swabs were collected daily and placed in 2 mL EP tubes containing 1 mL of sterile normal saline, and stored at −80 °C until future analysis. All goslings were euthanized by intravenous injection of pentobarbital sodium at 5 dpi, and the kidneys, liver, spleen, and cecal tonsils were collected from each gosling. Tissue samples were stored at −80 °C, and nucleic acids were extracted to quantify the viral copy numbers.

In order to verify whether the viral copy numbers in various tissues of goose embryos were consistent with those in the tissues of goslings, fifteen 12-day-old healthy goose embryos were similarly divided into three groups (five embryos per group). The first group was inoculated with 0.2 mL of the GDYJ-21-01 strain (suspension containing a virus load equal to 5.0 × 10^9^ copies) at the allantoic membrane per embryo; the second group received 0.2 mL of the GDZJ-21-01 strain (suspension containing a virus load equal to 5.0 × 10^9^ copies); and the third group was inoculated with 0.2 mL of sterile saline. At 5 dpi, the embryoid body was removed, and the allantoic fluid, lungs, kidneys, and intestinal tissues were collected separately from each embryo. Samples were stored at −80 °C until subsequent nucleic acid extraction to determine viral copy numbers.

### 2.8. Quantitative RT-PCR (qRT-PCR) Analysis

The qRT-PCR products were inserted into the pMD18-T vector to construct plasmid standards containing the target fragment, following the method by Yuan et al. [31,32,33,34]. Briefly, viral nucleic acids of GAstV-I and GAstV-II were extracted from the GDYJ and GDZJ strains using the Virus Nucleic Acid Extraction Kit (TIANLONG, Xi’an, China). The target fragments were amplified by qRT-PCR using qRT-PCR detection primers (Table 1). The qRT-PCR products were purified using the Gel Extraction Kit (Omega Bio-Tek, Inc., Norcross, GA, USA), ligated into the pMD18-T vector, and transformed into DH5α-competent cells. After selection on LB agar containing ampicillin, positive colonies were identified by DNA sequencing of the target gene. Plasmids were extracted using the Plasma Midi Kit (Omega Bio-Tek, Inc.), and plasmid concentration was converted to copy number according to the method of Yi et al. [34]. qRT-PCR was conducted using a HiScript II One Step qRT-PCR SYBR Green Kit (Vazyme, Nanjing, China) and a LightCycler 96 Real-Time PCR System (Roche, Basel, Switzerland). Viral replication was expressed as viral RNA copies/µL. The qRT-PCR primers used for detection are detailed in Table 1.

The limit of quantification and detection of the qRT-PCR assay was evaluated using serial 10-fold dilutions of pMD18-GAstV-I and pMD18-GAstV-II, respectively (the initial copy number of pMD18-GAstV-I was: 9.61 × 10^10^ copies/μL, and that of pMD18-GAstV-II was: 9.31 × 10^10^ copies/μL). Results were interpreted as ‘positive’ when Cq ≤ 35 and ‘negative’ when Cq > 35. The specificity of the qRT-PCR assay was evaluated using other astroviruses, such as duck astrovirus (DAstV), which was preserved in our laboratory [35]. Each experiment was repeated at least three times to ensure reproducibility.

### 2.9. Comparison of GAstV Capsid Structures

The AlphaFold 2.0 online platform [36] (https://colab.research.google.com/github/sokrypton/ColabFold/blob/main/AlphaFold2.ipynb (accessed on 5 January 2023)) was used to conduct structural analyses of the GAstV capsid proteins. For protein 3D structure prediction, FASTA files containing the amino acid sequences of the two GAstV ORF2 proteins (determined from the DNA sequences in this study) were submitted. Default operating parameters were used during the prediction, with the running mode set to monomer. AlphaFold 2.0 provides five independent models post-prediction, and the model with the highest predicted internal distance-based threshold (IDDT) score is generally regarded as the most accurate representation. Thus, the model with the highest score was selected for subsequent comparative analysis. The predicted 3D structures were characterized using PyMOL (Version 2.5.0) software [37]. Alignment of ORF2 amino acids from the two GAstV subtypes was performed using the sequence alignment function in Discovery Studio software (Discovery Studio 3.5, Accelrys, Co., Ltd., San Diego, CA, USA).

### 2.10. Statistical Analysis

Data were processed by GraphPad Prism 5.0 (GraphPad Software Inc., San Diego, CA, USA). The data are presented as the mean ± standard error of the mean (SEM). The significance of differences between two groups was determined with tow-tailed unpaired Student’s *t*-test. A *p*-value of less than 0.05 was considered significant (* *p* < 0.05, ** *p* < 0.01, *** *p* < 0.001, **** *p* < 0.0001).

## 3. Results

### 3.1. Clinical Features, Sample Identification and Histopathological Analysis

**Figure 1:**
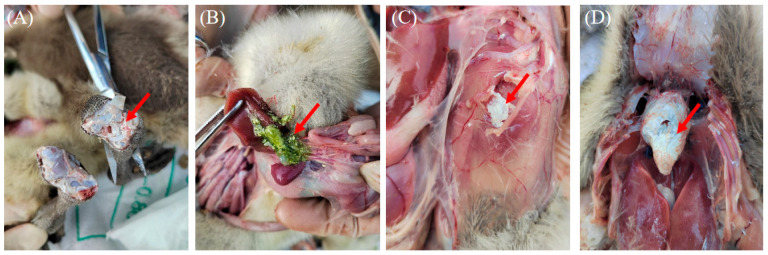
Clinical symptoms of gout disease in geese. (**A**) Urate deposition at the joints of the leg bones. (**B**) Urate crystals in the gallbladder. (**C**) Subcutaneous urate deposition. (**D**) Urate deposition on the surface of the heart.

Goslings infected with GAstV typically exhibit symptoms such as mental depression, anorexia, and reduced mobility. Necropsy findings of goslings in this study revealed a significant accumulation of urate crystals in affected geese, particularly in the heart, joints, gallbladder, and rib sidewalls (Figure 1).

All 385 goose gout samples tested positive for GAstV via RT-PCR. Among the samples, 9.6% (37/385) were solely infected with GAstV-I, while 40.0% (154/385) were infected exclusively with GAstV-II. The remaining 50.4% (194/385) were co-infected with GAstV-I and GAstV-II. These results indicate that GAstV-II is the primary source of infection, and co-infection with GAstV-I and GAstV-II is more common in geese. Among the 385 GAstV-positive samples, we also tested for other common viral infections in geese (Supplementary Table S1). The infection rate of GAstV with other single viruses was 50.7%, with dual viral infections was 24.4%, and with triple viral infections was 5.5%, indicating that GAstV infection is frequently associated with mixed infections involving other viruses.

**Figure 2:**
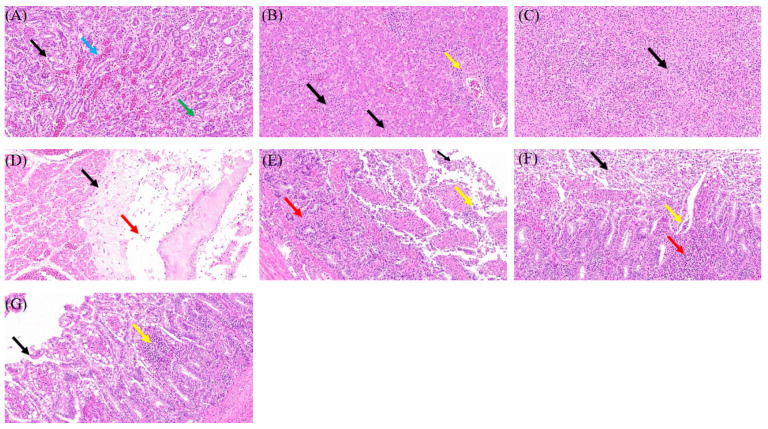
H and E staining of tissue sections from geese with gout in clinical samples (×200). (**A**) Kidney. (**B**) Liver. (**C**) Spleen. (**D**) Heart. (**E**) Intestinum tenue. (**F**) Cecal tonsil. (**G**) Colon. The arrow indicates the lesion area.

Histopathological analysis via H and E staining demonstrated multiple instances of tubular epithelial cell shedding in renal tissue (black arrow), eosinophilic clumps within the renal tubule lumens (green arrow), and localized venous blood stasis (blue arrow) (Figure 2A). In liver tissue, scattered hepatocyte necrosis (black arrow) and nuclear fragmentation or dissolution were observed, alongside extensive lymphocyte infiltration surrounding the liver lobules and portal areas (yellow arrow) (Figure 2B). Spleen tissue exhibited relatively few lymphocytes, with rare parenchymal cell necrosis (black arrow) and nuclear fragmentation (Figure 2C). In heart tissue, a substantial eosinophilic exudate was noted beneath the epicardium (black arrow), accompanied by punctate infiltration of lymphocytes and granulocytes (red arrow) (Figure 2D). In the small intestine, numerous epithelial cells were shed from the mucosal layer (black arrow), with increased epithelial cell necrosis (yellow arrow) and nuclear fragmentation. Additionally, localized connective tissue proliferation was observed in the lamina propria (red arrow), with widening of the distance between intestinal glands (Figure 2E). Similarly, a significant number of epithelial cells were shed from the cecal mucosal layer (black arrows), with increased epithelial cell necrosis (yellow arrows) and nuclear fragmentation, and localized infiltration of lymphocytes and granulocytes in the submucosal layer (red arrows) (Figure 2F). Comparable findings were noted in the ileal tissue (black arrows), with minor infiltration of lymphocytes and granulocytes in the lamina propria (yellow arrows) (Figure 2G). The pathological changes mentioned above were not observed in the tissues of the control goslings (Supplementary Figure S1).

### 3.2. Virus Isolation, Whole Genome Sequencing, and Recombination Analysis

Clinical samples exclusively infected with GAstV-I or GAstV-II (excluding other common goose viruses) were subjected to continuous passage for five generations in 11-day-old goose embryos. After virus identification via PCR or RT-PCR and the exclusion of infections with other common goose viruses, two GAstV strains were successfully isolated. Whole genome sequencing was then performed.

Sequencing results indicated that the GDYJ strain possessed a total genome length of 7299 bp, with a nucleotide similarity of 98.4% to the classic AHDY (MH410610.1) strain, both of which are classified as GAstV-I. Conversely, the GDZJ strain had a genome length of 7183 bp, with a nucleotide similarity of 97.9% with the classic GD (MG934571.1) strain, which is classified as GAstV-II. The whole-genome similarity between the GDYJ and GDZJ isolates is 54.0%, while the ORF2 gene similarity is 50.7%. Both GDYJ and GDZJ strains exhibited the typical astrovirus gene arrangement of 5′-UTR-ORF1a-ORF1b-ORF2-3′ UTR. The genome sequences of the two strains have been submitted to NCBI and assigned the corresponding GenBank accession numbers (GDYJ-21-01: OP776630.1; GDZJ-21-01: OP776629.1).

Although no recombination events were identified in the GDYJ strain itself, recombination analysis revealed that a recombination event occurring between nucleotides 2459–3430 in the genome of the JSXZ (OR827024.1) strain primarily originated from the FLX (NC_034567.1) strain and secondarily from the GDYJ strain, with a *p*-value of 1.410 × 10^−15^ (Supplementary Figure S2A,B). These results suggest that the GDYJ strain may act as a minor parental donor contributing genetic material to other GAstV strains. Analysis of recombination events in the GDZJ strain revealed two recombination breakpoints at nucleotide positions 1–838 and 7098–7152 in the full-length genome (Supplementary Figure S2C,D). The major parental strain was identified as SD03 (OP621341.1), while the minor parental strain was 1812LMG (MN127959.1), with a *p*-value of 5.53 × 10^−14^.

### 3.3. Phylogenetic Analysis of GAstVs

To elucidate the phylogeny of the two isolated GAstVs within the broader astrovirus family, we searched the NCBI Nucleotide database using the keywords, ‘Astrovirus and complete genome’ and received 844 results (search date: 17 August 2024).

**Figure 3:**
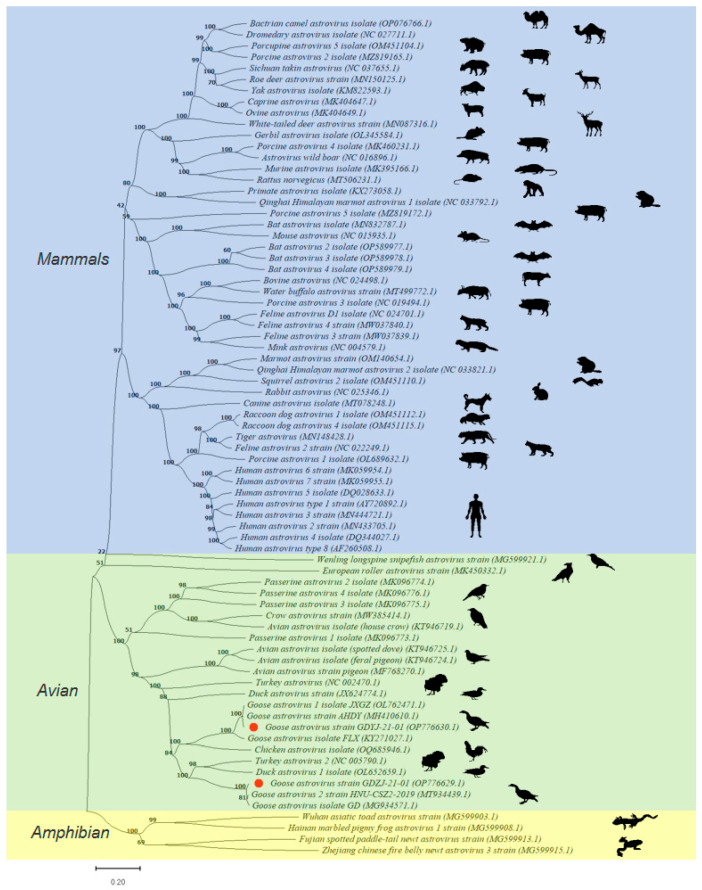
Evolutionary relationships between astroviruses. The evolutionary history was inferred using the Neighbor-Joining method. The percentage of replicate trees in which the associated taxa clustered together in the bootstrap test (1000 replicates) is shown above the branches. The tree is drawn to scale, with branch lengths in the same units as those used to infer the evolutionary distances. The evolutionary distances were computed using the Maximum composite likelihood method and are expressed as the number of base substitutions per site. This analysis included 75 nucleotide sequences, with all ambiguous positions removed for each sequence pair (pairwise deletion option). Evolutionary analyses were performed in MEGA11. Red dots represent the strains isolated in this study.

A total of 75 representative genome-wide strains were selected based on species and subtypes, including 48 from mammals (blue background), 23 from poultry (green background), and four from amphibians (yellow background) (Figure 3). Phylogenetic analysis (Figure 3) revealed that the GDYJ and GDZJ strains belong to distinct populations of GAstVs, separated by astroviruses from chickens, turkeys, and ducks. Thus, the findings suggest that the two subtypes may have different evolutionary origins.

The major branches of the evolutionary tree indicate a very low likelihood of avian astroviruses infecting mammals and amphibians, highlighting the presence of an evolutionary barrier. However, there is a significant potential for cross-species infection among poultry, particularly involving geese, chickens, turkeys, and ducks. Co-infection with GAstV-I and GAstV-II may increase the likelihood of viral recombination, thereby heightening the risk of cross-species infection in poultry. Further investigations are warranted to ascertain whether similar phenomena exist among other avian astroviruses.

### 3.4. Difference in the Tissue Tropism of GAstVs

To evaluate the sensitivity and specificity of the qRT-PCR assay, standard curves were first established. For GAstV-I (GDYJ), plasmid standards ranging in concentration from 9.61 × 10^9^ to 9.61 × 10^0^ copies/μL were subjected to 10-fold serial dilutions to construct a standard curve of copy number versus cycle threshold (Cq) value (Supplementary Figure S3A,B). The resulting equation was Y = −3.2443X + 41.95, with a correlation coefficient (R^2^) of 0.99, and the detection limit was 9.61 × 10^1^ copies/μL. Similarly, for GAstV-II (GDZJ), plasmid standards ranging from 9.31 × 10^9^ to 9.31 × 10^0^ copies/μL were used to generate a standard curve (Supplementary Figure S3C,D). The linear regression equation was Y = −3.2115X + 42.24, with an R^2^ value of 1.00, and a detection limit of 9.31 × 10^1^ copies/μL.

As shown in Supplementary Figure S4, the specificity of the assay was evaluated using plasmids pMD-18-GAstV-I and pMD-18-GAstV-II, DAstV viral nucleic acid, and nuclease-free water as templates. When GAstV-I-specific primers were used, only the GAstV-I plasmid yielded a positive amplification signal (red curve). Conversely, when GAstV-II-specific primers were employed, only the GAstV-II plasmid was amplified (green curve), indicating high assay specificity without cross-reactivity to DAstV.

**Figure 4:**
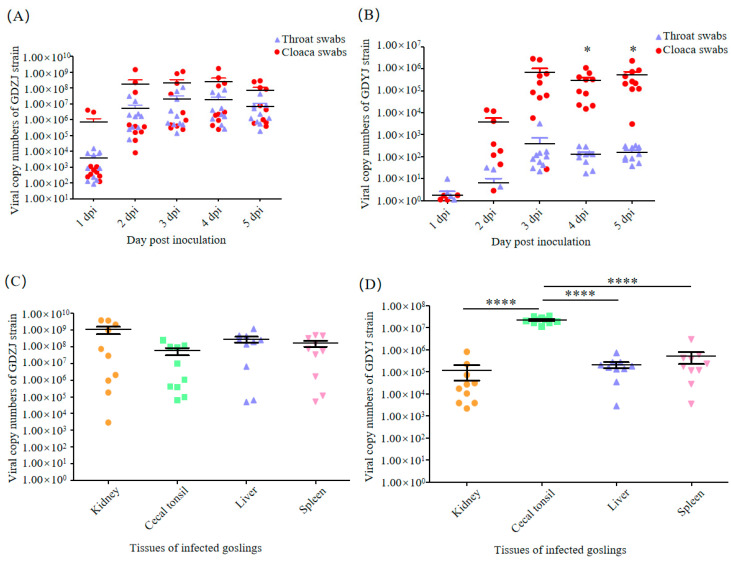
Viral copy numbers in goslings infected with GAstV. Viral copy numbers in throat and cloacal swabs of goslings infected with (**A**) the GDZJ strain or (**B**) the GDYJ strain; viral copy numbers in gosling tissues infected with (**C**) the GDZJ strain or (**D**) the GDYJ strain at 5 dpi. Each dot represents data from one gosling (n = 10). All assays were performed in triplicate. Data are presented as the mean ± SEM. * *p* < 0.05, **** *p* < 0.0001.

For the purpose of identifying the differences in host infection between the two subtypes of GAstVs strains, the GDZJ and GDYJ strains isolated in this study were employed to infect 3-day-old goslings and 12-day-old goose embryos, respectively. The results illustrated in Figure 4A indicate that virus in throat and cloacal swabs from geese infected with the GDZJ strain was detectable at 1 dpi, peaking at 2 dpi, followed by stable viral shedding from 3 dpi to 5 dpi, with no significant difference between throat and cloacal swabs. In contrast, Figure 4B shows that after infection with the GDYJ strain, only a few geese shed the virus at 1 dpi, whereas all geese exhibited viral shedding by 3 dpi, and maintained a consistent level of shedding until 5 dpi. Notably, a significant difference was observed in viral shedding between throat and cloacal swabs for the GDYJ strain, with cloacal swabs yielding much higher viral loads (Figure 4B).

Following the collection of throat and cloacal swabs at 5 dpi, the geese were euthanized for dissection. No overt symptoms, such as urate crystallization, were observed in the organs. Typical diseased organs were collected and viral copy numbers were quantified. As depicted in Figure 4C,D, viral copy numbers in the kidneys, cecal tonsils, liver, and spleen of goslings infected with the GDZJ strain showed no significant differences. Given that the goslings were not SPF (specific pathogen-free), individual physiological health states varied, resulting in poor parallelism in the group. Interestingly, viral copy numbers in the cecal tonsils of geese infected with the GDYJ strain were significantly higher than those in the kidneys, liver, and spleen, with no significant differences among the latter three organs. Overall, the findings suggest a strong preference of the GDYJ strain for intestinal tissue. At the same time, the GDZJ strain has a broad tropism towards all tissues tested.

**Figure 5:**
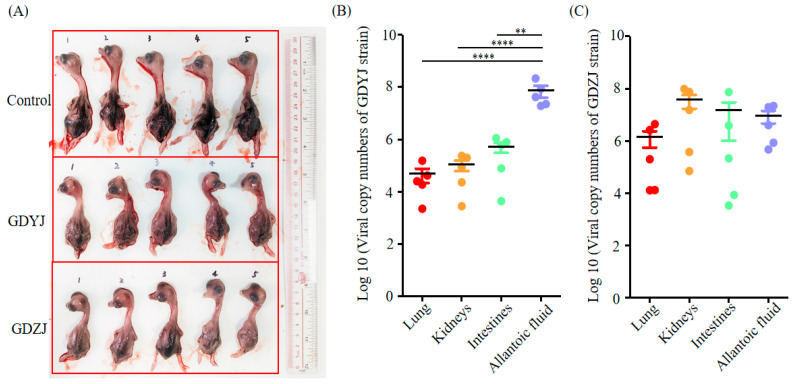
Goose embryos infected with GAstV at 5 dpi. (**A**) Morphology of goose embryos at 5 dpi after inoculation with physiological saline (control), the GDYJ strain, or the GDZJ strain in the allantoic cavities. Viral copy numbers in different tissues and allantoic fluid of goose embryos inoculated with (**B**) the GDYJ strain or (**C**) the GDZJ strain. Data were analyzed using GraphPad Prism 5.0. The number of embryos per group was five. All assays were performed in triplicate. Data are presented as the mean ± SEM. ** *p* < 0.01, **** *p* < 0.0001.

In order to verify the results of in vivo infection and confirm the infectivity of the virus, the GDYJ and GDZJ strains isolated in this study were used to infect 12-day-old goose embryos, respectively. At 5 dpi, the embryos were harvested for comparison. Compared to the virus-inoculated groups (Figure 5A), control group embryos exhibited superior growth and development, with larger individual sizes and thicker back feather growth. Both GAstV subtypes inhibited the growth and development of goose embryos. Figure 5B reveals that viral copy numbers in the allantoic fluid of embryos infected with the GDYJ strain were significantly higher than those in lung, kidney, and intestinal tissues. Figure 5C illustrates that there were no significant differences in viral copy numbers among the allantoic fluid, lungs, kidneys, and intestinal tissues of embryos infected with the GDZJ strain. In addition, no GAstV subtypes were detected in the control group samples during the gosling and embryonic virus infection experiments.

### 3.5. Structural Comparison of GAstV Capsids

The ORF2 protein of astrovirus is a viral capsid protein that plays a pivotal role in the viral infection process within the host. Although both subtypes of GAstV can infect goslings and cause gout symptoms, compared to GDYJ strain, GDZJ strain has a wider tissue tropism and stronger viral replication ability. Consequently, we compared the ORF2 protein structures of the two GAstV strains.

**Figure 6:**
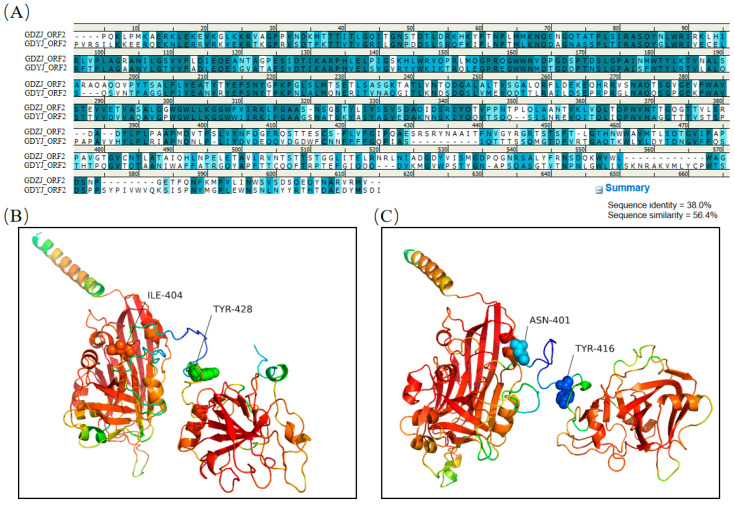
Structural analysis of ORF2 proteins from the GDZJ and GDYJ strains. (**A**) Amino acid sequence alignment of the ORF2 protein between the GDZJ and GDYJ strains. (**B**) Structural prediction of the ORF2 protein for the GDYJ strain. (**C**) Structural prediction of the ORF2 protein for the GDZJ strain.

Initially, five independent models were evaluated based on the AlphaFold2 prediction results. Supplementary Figure S5 displays the predicted IDDT scores for the ORF2 proteins of both subtypes, which positively correlated with the confidence levels indicated by the models. Generally, a score exceeding 90 is considered indicative of very high precision, while a score greater than 70 suggests a reasonably accurate backbone prediction. A score below 50 indicates an unreliable random position. Based on the evaluation scores presented in Supplementary Figures S5–S7, two rank_1 prediction models for ORF2 subtypes were selected for subsequent structural analysis. The amino acid comparison results for ORF2 from GDYJ and GDZJ (Figure 6A) indicated a sequence identity of 38.0% and a sequence similarity of 56.4%. The 3D structural representations of ORF2 proteins from the GDYJ strain (Figure 6B) and GDZJ strain (Figure 6C) illustrated that the two proteins could be divided into two structural regions, connected at positions I404-T428 and A401-T416, respectively.

**Figure 7:**
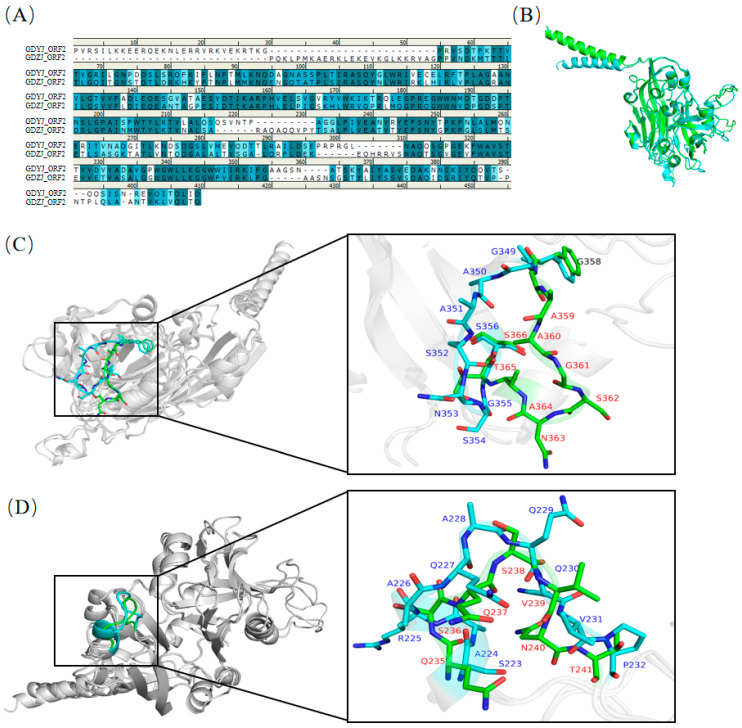
Analysis of structural differences in the ORF2-VP34 region between the GDYJ and GDZJ strains. (**A**) Amino acid alignment of the ORF2-VP34 region proteins between the GDYJ and GDZJ strains. (**B**) Overlay diagram of the VP34 region structures for the GDYJ and GDZJ strains. Green represents the GDYJ strain, while blue represents the GDZJ strain. (**C**) Significant structural differences are observed between G349-S356 of the ORF2-VP34 region in the GDZJ strain and G358-S366 of the ORF2-VP34 region in the GDYJ strain, due to the absence of amino acids A and G in the GDZJ strain. (**D**) Additional structural differences are noted between S223-P232 of the ORF2-VP34 region in the GDZJ strain and Q235-T241 of the ORF2-VP34 region in the GDYJ strain. S223-A226 of the ORF2-VP34 region in the GDZJ strain forms an α-helix, whereas Q235-Q237 of the ORF2-VP34 region in the GDYJ strain forms a loop. The blue font denotes amino acid abbreviations and positions for the GDZJ strain, while the red font denotes those for the GDYJ strain.

Through amino acid sequence alignment (Figure 7A), it was found that the sequence similarity of the first half of the VP34 region between the two proteins was as high as 70.0%. Subsequent structural overlap comparisons revealed (Figure 7B) two regions with significant structural differences. A structural overlap comparison of the ORF2-VP34 region between the two strains (Figure 7C) indicated substantial overlap in the overall structure. Notably, the G349-S356 segment of the ORF2 region of the GDZJ strain and the G358-S366 segment of the ORF2 region of the GDYJ strain exhibited significant differences that were attributable to the absence of one alanine and one glycine in GDZJ ORF2, thus resulting in structural discrepancies.

**Figure 8:**
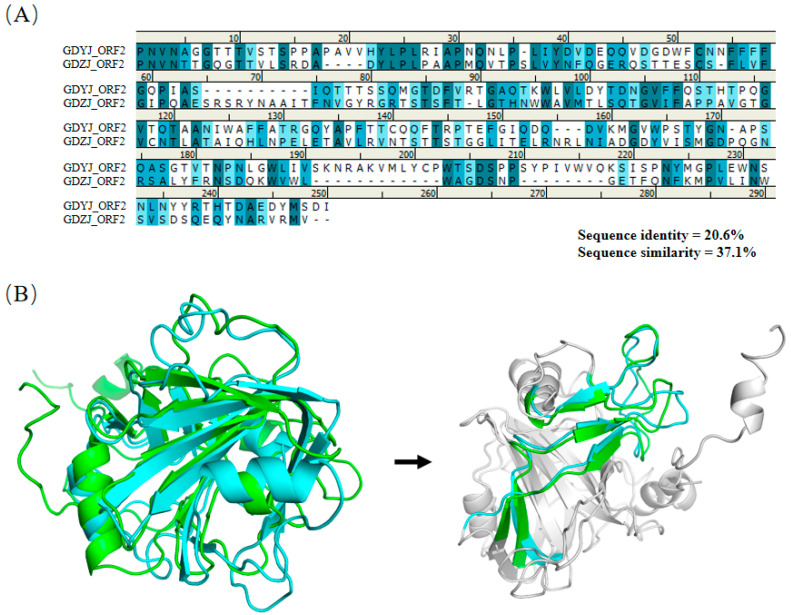
Analysis of structural differences in the ORF2-VP27 region between the GDYJ and GDZJ strains. (**A**) Amino acid alignment of the ORF2-VP27 region proteins between the GDYJ and GDZJ strains. (**B**) Overlay diagram of the VP27 region structures for the GDYJ and GDZJ strains. Green represents the GDYJ strain, while blue represents the GDZJ strain. The colored area on the right highlights five β-sheet structures with high similarity between the two proteins.

Significant structural differences were observed between S223-P232 of the ORF2 region of GDZJ and Q235-T241 of the ORF2 region of GDYJ (Figure 7D), wherein S223-A226 of GDZJ ORF2 forms an alpha helix, while Q235-Q237 of GDYJ ORF2 forms a loop region. The amino acid similarity in the VP27 region of the C-terminus of the two proteins was low (Figure 8A), with a sequence similarity of 37.1% and sequence identity of 20.6%. Nevertheless, structural alignment revealed that the L430-E456 and H484-A513 sequences of GDZJ-ORF2 exhibited high structural similarity with the L441-S468 and Q487-A516 segments of GDYJ-ORF2 containing five β-sheets. These structural differences may be the reasons for the differences in tissue tropism and replication ability between different subtypes of GAstV.

## 4. Discussion

An increasing body of evidence indicates that both GAstV-I and GAstV-II can cause gout symptoms in geese [6,13,38,39]. This study analyzed samples from geese exhibiting gout symptoms collected between 2019 and 2021 in Guangdong Province, China. Compared to the data collected by our team from 2022 to 2024, it is evident that since the discovery of GAstV, GAstV-II infections have emerged as the primary causes of gout in goslings in the Guangdong region, with a detection of 40% to 42.4%, while GAstV-I has not been detected. Although the incidence rate of GAstV-I infection alone is decreasing (with detection rates of 9.6% in this study and 5.0% in Xiang et al.), the prevalence of mixed infections involving both GAstV-I and GAstV-II is increasing (with detection rates of 50.4% in this study and 52.6% in Xiang et al. [15]). Furthermore, co-infections exceeding 40.0% have also been reported in other provinces [6,40]. Among the 385 samples of goose gout analyzed in the current study, co-infection of GAstV with other viruses—including GPV, GHPV, TMUV, FAdV, GoCV, and GRV—was observed in over 80.0% of samples, excluding co-infections involving the two subtypes of GAstVs (Supplementary Table S1). This suggests that GAstV infections often occur in complex viral ecosystems, potentially influencing disease progression. Exploring potential interactions—such as competition, synergy, or recombination—would require time-course studies and controlled co-infection models, which we plan to investigate in future research. Due to our lack of proactive monitoring of GAstV infections in geese without gout, we cannot ascertain whether GAstV has developed into a conditional infection or whether there are differences in pathogenicity. Thus, our future research will enhance the active surveillance of GAstV to further elucidate its infectious characteristics.

Currently, there is no literature or other evidence confirming that GAstV can infect wild birds. However, a substantial body of research indicates that GAstV can cross species barriers to infect poultry, including chickens and ducks [10,41,42]. In the evolutionary tree presented in Figure 3, it is evident that, in addition to chickens and ducks, turkeys are also at risk of GAstV infection. Given the inherent instability of RNA viruses, it is possible that a high prevalence of mixed infections involving GAstV-I and GAstV-II may increase the likelihood of GAstV recombination or mutation, thereby enhancing the potential for cross-species transmission.

Following infection with the GDZJ strain, viral copy numbers in throat and cloacal swabs rapidly peaked (Figure 4A). After a host is infected, the virus replicates explosively and maintains a prolonged horizontal plateau phase. This phenomenon aligns with the self-limiting effect of high 2′-5′oligoadenylate synthetase-like gene expression induced by GAstV ORF2-mediated innate immunity in the host, as described by Ren et al. [20]. Furthermore, this observation is consistent with the growth kinetics of GAstV-II in primary kidney tubular epithelial cells derived from geese [43]. Thus, such findings may indicate why acute outbreaks of gout symptoms frequently occur in association with GAstV-II during the process of raising geese.

Since the identification of astroviruses, the primary symptoms of infection in mammals have been enteritis or diarrhea [44,45], with rare strains causing neurological diseases [46]. However, most reports of GAstVs primarily focus on gout symptoms in the host, with limited documentation on intestinal symptoms, such as diarrhea. A recent study indicated that GAstV infection resulted in a decrease in beneficial bacteria and an increase in potentially pro-inflammatory bacteria within the gut microbiome of goslings [47]. Although the current study did not determine whether infection led to diarrhea in goslings, the data suggest that GAstV infection adversely affects gut health.

The GDYJ strain exhibited slow replication in the host (Figure 4), with viral copy numbers becoming detectable in most throat swabs by 3 dpi. Notably, viral copy numbers in cloacal swabs were significantly higher than those in throat swabs (Figure 4B), and viral copy numbers in cecal tonsils were significantly greater than those in the kidney, liver, and spleen (Figure 4D). Conversely, the GDZJ strain did not exhibit this phenomenon, suggesting a broader tissue tropism. We analyzed GAstV infection experiments in studies published on the NCBI website, which indicated that the low infection rate of GAstV-I alone, or due to the absence of a suitable in vitro infection model for GAstV-I, has resulted in limited data being available regarding GAstV-I isolation and infection in goslings.

Research on tissue tropism following GAstV-II infection has primarily identified the kidney, liver, and spleen as the tissues with the highest viral copy numbers [26,48,49,50]. To validate this observation, goose embryos were separately inoculated with the GDYJ (GAstV-I) and GDZJ (GAstV-II) strains. The results revealed that viral copy numbers in the allantoic fluid of embryos infected with the GDYJ strain were significantly higher than those in the lung, kidney, and intestinal tissues. In contrast, no significant differences were observed among the tissues infected with the GDZJ strain. Given the developmental stage of the goose embryos, only the lungs, kidneys, and intestinal tissues could be isolated during dissection. Notably, allantoic fluid, as a partial excretory product of the goose embryo, may serve a role analogous to the intestinal contents of goslings. If this is the case, it suggests that the gut, or its microbiota, could be the primary environment where the GDYJ strain thrives. This hypothesis may also explain why GAstV-I fails to replicate in LMH cells, whereas GAstV-II does.

Previous studies have demonstrated that GAstV viral loads in tissues peak around 5 days post-infection (dpi) [5,26]. Our previous research further confirmed that the acute phase of GAstV infection typically occurs within this timeframe [24]. Therefore, to investigate early pathological changes and viral dynamics, this study selected 5 dpi as the endpoint of the infection experiment. This timing aims to provide insights into potential intervention strategies for preventing gosling gout before the onset of severe clinical symptoms.

A study conducted by our team confirmed that GAstV-I was prone to replication in goose embryos, but not in LMH cells, whereas GAstV-II exhibited the opposite trend [15]. Coincidentally, Wei et al. [6] recently investigated tissue tropism using the GAstV-I strain (JSXZ), and found that the highest viral copy numbers were present in the kidneys and intestines following infection. Those findings align with our data. Our subsequent studies will further refine our analysis of different intestinal segments to determine whether variations in viral content are attributable to specific regions or the gut microbiota. Due to the lack of SPF goslings, individual and batch variations among goslings were unavoidable and may account for some of the differences observed in specific populations within this study. These variations also reflect the realities of commercial goose farming, where goslings from the same flock infected with GAstV may exhibit differing degrees of gout symptoms despite exposure to the same pathogen. Additionally, the sample size in this study was relatively limited, and only one representative strain was used for each GAstV subtype. Therefore, the findings cannot be fully generalized to all GAstV-I and GAstV-II strains. In future studies, we aim to increase the sample size and include geese of different breeds and from diverse geographic regions for comparative analysis.

Given the significant differences in tissue tropism between GAstV-I and GAstV-II, this study analyzed the similarities and differences in the capsid protein structures of the two subtypes of GAstVs. Based on the structure of human astrovirus, the first 400 amino acids of the GAstV ORF2 N-terminus correspond to VP34, while the C-terminus comprises 300 amino acids of VP27 [23]. VP27 is partially cleaved into VP25 by a protease, and both VP25 and VP27 form a dimeric spike structure on the astrovirus capsid, which may play a role in host receptor recognition [51]. By comparing the secondary structures of amino acids, we demonstrated that the VP34 sequences of the two subtypes exhibited high similarity (Figure 6A), indicating that the VP34 sequences are relatively conserved. However, upon comparison of the overlap between the VP34 tertiary structure (Figure 7B), two significant regions of difference were identified (Figure 7C,D). One difference arose from the structural inconsistency due to the absence of two amino acids (AG) in GAstV-II (Figure 7C), while the other resulted from the formation of distinct domains attributed to variations in amino acid sequences (Figure 7D). The structural differences may affect the conditions under which viral capsids release viral nucleic acids into the host. The structural alignment of VP27 confirmed that, although both strains of GAstV possess five β-sheet structures in their VP27 proteins (Figure 8B), there are substantial differences in their amino acid sequences (Figure 8A). For both GAstV and human astrovirus, the amino acid sequences that interact with neutralizing antibodies reside within the VP27 region [21,52,53]. While the tertiary structure similarity of the VP27 protein between the two GAstV subtypes maintains comparable viral morphology, significant differences in amino acid sequences lead to variations in host infection processes. In future studies, we will employ reverse genetic technology to exchange different regions between the two subtypes to determine the causes of differences in tissue tropism, thereby providing a theoretical basis for vaccine development. Additionally, we intend to employ X-ray crystallography or low-temperature electron microscopy (EM) for more precise structural analysis.

## 5. Conclusions

In this study, we successfully isolated GDYJ (GAstV-I subtype) and GDZJ (GAstV-II subtype) strains and evaluated their growth characteristics both in vivo and in ovo. Our findings demonstrated that GDZJ exhibited a broader tissue tropism compared to GDYJ. Through tertiary structure analysis of the ORF2 proteins from both virus subtypes, we identified key structural similarities and differences. Notably, the structural divergence and low sequence similarity in the VP27 region of GDYJ and GDZJ ORF2 proteins may contribute to their distinct tissue tropisms. These findings provide important evidence supporting the differential tissue tropism of GDYJ and GDZJ strains, emphasizing the need for further research into the antigenic variations between these subtypes.

## Figures and Tables

**Table 1:** Primers used in this study for amplification.

Primer Name	Primer Sequences (5′-3′)	Product (bp)	Purpose
GAstV-I ORF2-F	AGTGGAGTGTTATGCCTACCAGGAGA	2314 bp	Amplification of ORF2 gene by RT-PCR
GAstV-I ORF2-R	TTTTCCTCCGTAATCAGGAGGTTTTTTGGG		
GAstV-II ORF2-F	GCGAAAATGCACCCAATAGTGTTAGAGAG	2436 bp	Amplification of ORF2 gene by RT-PCR
GAstV-II ORF2-R	CCCCTGACCGGGTTTTTGTTTAAAGAAT		
GAstV-I-F	TCGTTGGGACCTGCAATATC	94 bp	Detection of viral RNA copies by qRT-PCR
GAstV-I-R	TAAAGAGACCACCAGCGAAAG		
GAstV-II-F	GGGTGATCCGCAAGGAAATA	98 bp	Detection of viral RNA copies by qRT-PCR
GAstV-II-R	AAGTTTCGCCAGGGTTAGAG		

Forward and reverse primers are indicated by F and R, respectively.

## Data Availability

The original contributions presented in this study are included in the article. Further inquiries can be directed to the corresponding author.

## Supplementary Materials

The following supporting information can be downloaded at https://www.mdpi.com/article/10.3390/microorganisms13051037/s1. Supplementary Figure S1: H and E staining of tissue sections from control goslings (×200). Supplementary Figure S2: Recombinant analysis of the GDYJ (OP776630.1) and GDZJ (OP776629.1) strains. Supplementary Figure S3: Standard amplification curves and sensitivity analysis of GAstV-I and GAstV-II. Supplementary Figure S4: Specificity analysis of qRT-PCR method. Supplementary Figure S5: Results of IDDT prediction. Supplementary Figure S6: Visualization of predicted aligned errors (PAE). Supplementary Figure S7: Number of sequences at each position. Supplementary Table S1: Mixed infections in 385 GAstV-positive samples with other viruses.
