# Supplementary material for: Isolation, Characterization, and Comparative Analysis of Two Subtypes of Goose Astrovirus in Guangdong Province, China
Source: Microorganisms. 2025 Apr 30;13(5):1037. doi: 10.3390/microorganisms13051037 (PMC12114045; doi:10.3390/microorganisms13051037)
Supplement: Supplementary file 1 [file microorganisms-13-01037-s001.zip › microorganisms-3524754 Supplementary Figure.pdf]

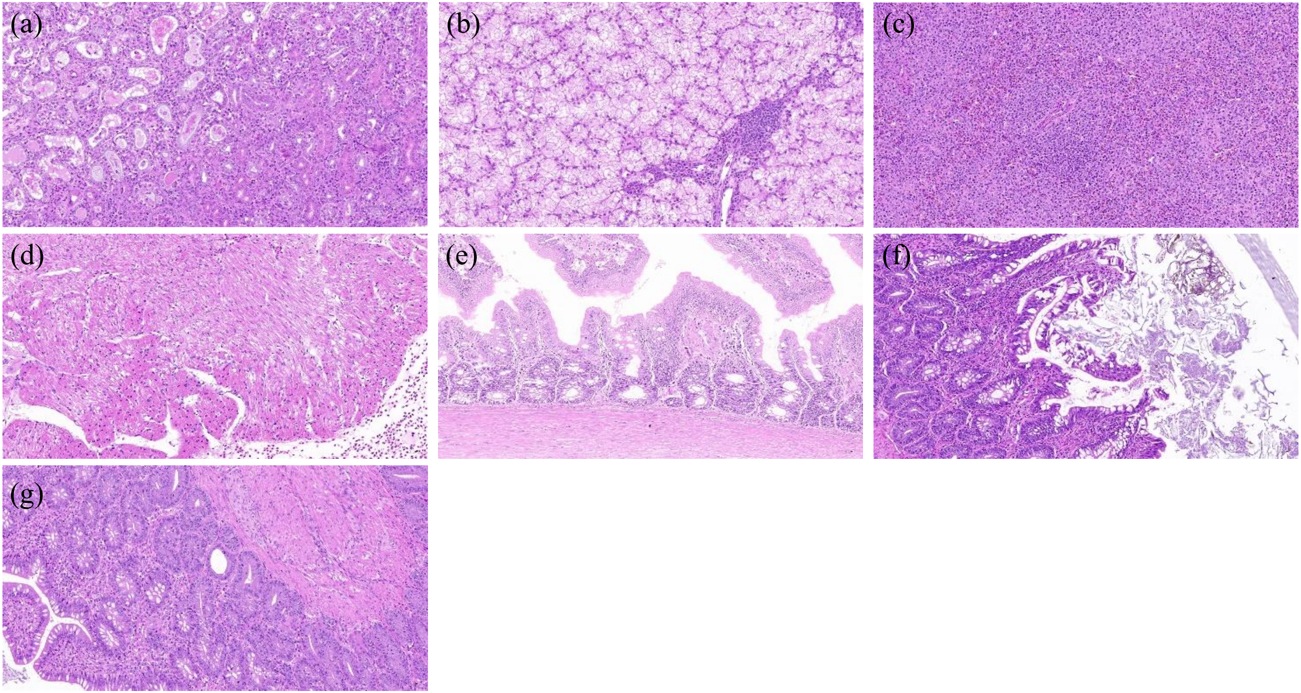

Supplementary Figure S1. H&E staining of tissue sections from control goslings (× 200). (a) Kidney; (b) Liver; (c) Spleen; (d) Heart; (e) Intestinum tenue; (f) Cecal tonsil; (g) Colon.

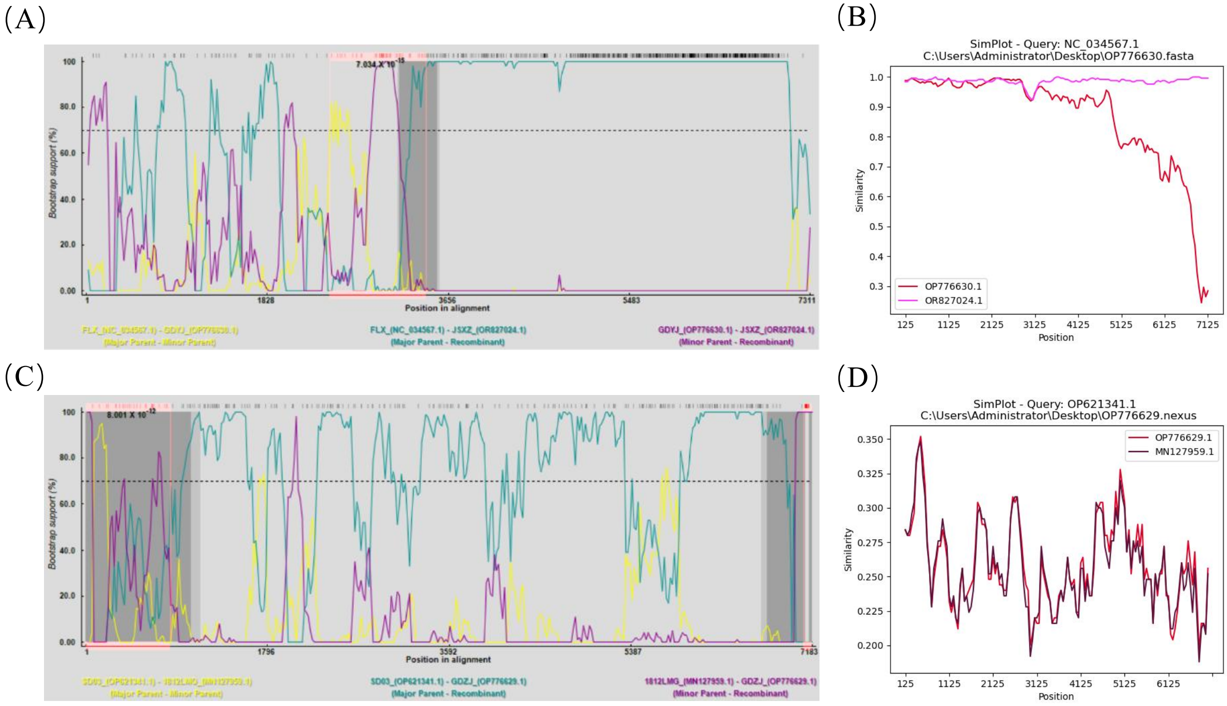

Supplementary Figure S2. Recombinant analysis of the GDYJ (OP776630.1) and GDZJ (OP776629.1) strains. The complete genome sequences of the (A-B) GDYJ, and (C-D) GDZJ strains were used for the recombination analysis by using the RDP4 (left) and Simplot software packages (right).

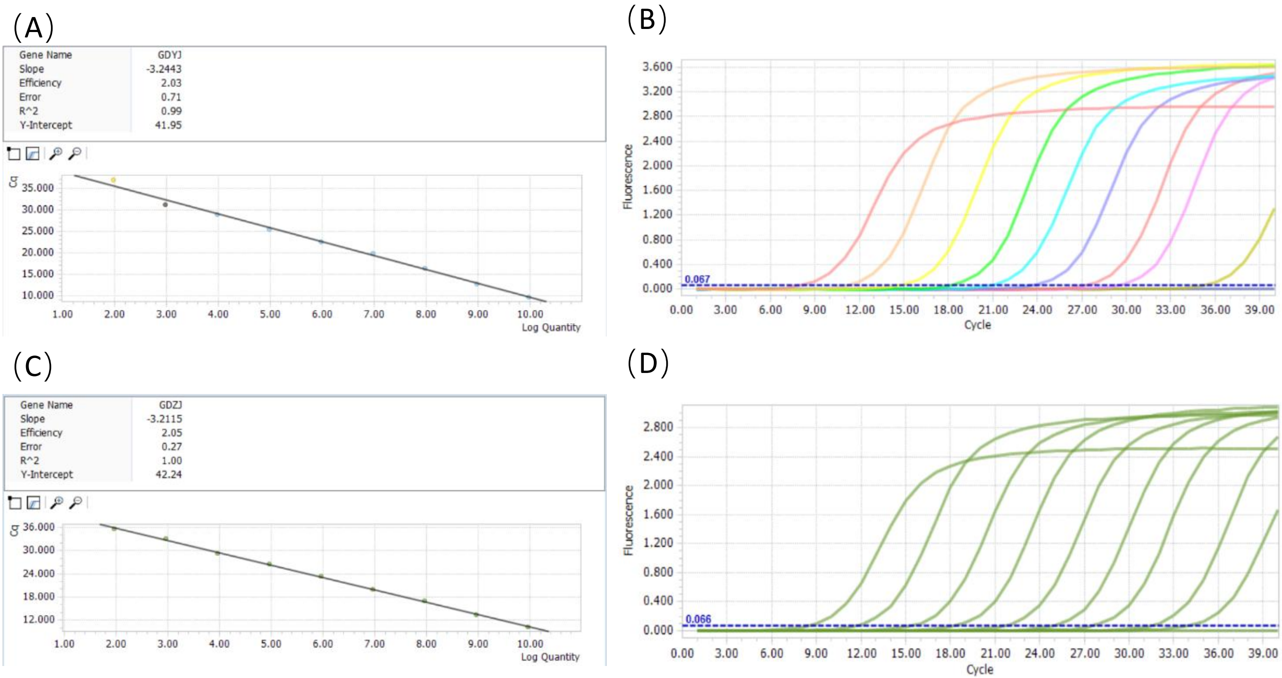

Supplementary Figure S3. Standard amplification curves and sensitivity analysis of GAstV-I and GAstV-II. Standard amplification curves for (A) GAstV-I and (C) GAstV-II. Sensitivity analysis of qRT-PCR method for detection of (B) GAstV-I and (D) GAstV-II.

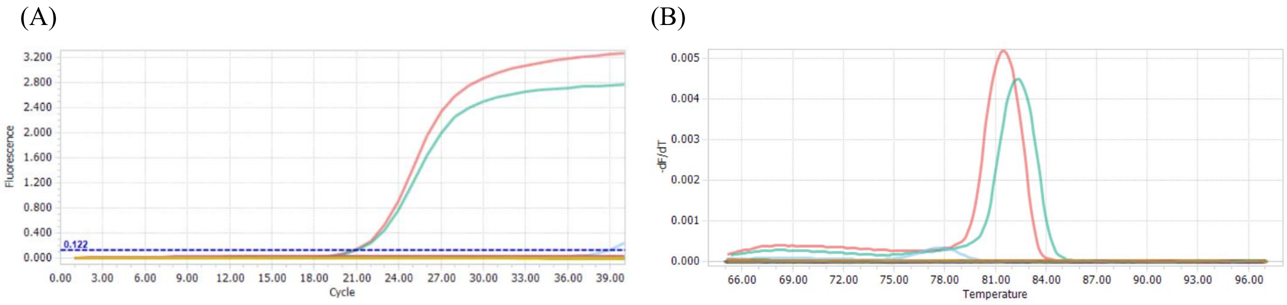

Supplementary Figure S4. Specificity analysis of qRT-PCR method for detection of GAstV-I (marked as red) and GAstV-I (marked as green) （A） Amplification curves, (B) Melting curves . Other curves represent unrelated viral and water controls.

(A) (B)

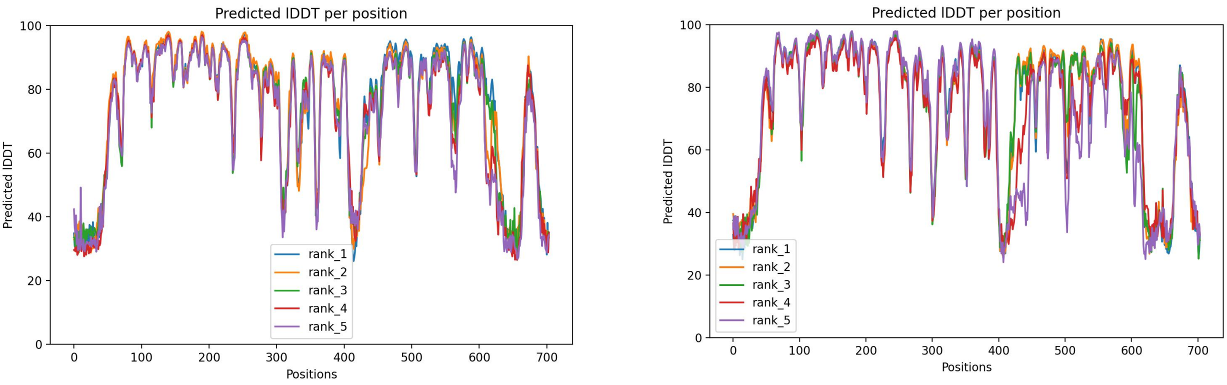

Supplementary Figure S5. Results of IDDT prediction. (A) GDYJ ORF2 protein; (B) GDZJ ORF2 protein. Rank_1 to Rank_5 are arranged in descending order of scores.

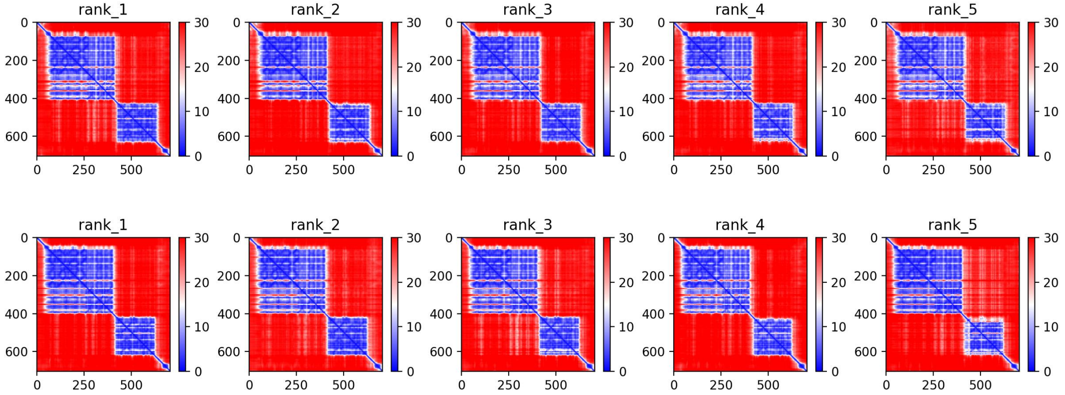

Supplementary Figure S6. Visualization of Predicted Aligned Errors (PAE). Identification of domains with relative positions and orientations for (A) GDYJ ORF2 and (B) GDZJ ORF2. The red area indicates a large relative error, while the blue area indicates a small relative error.

(A) (B)

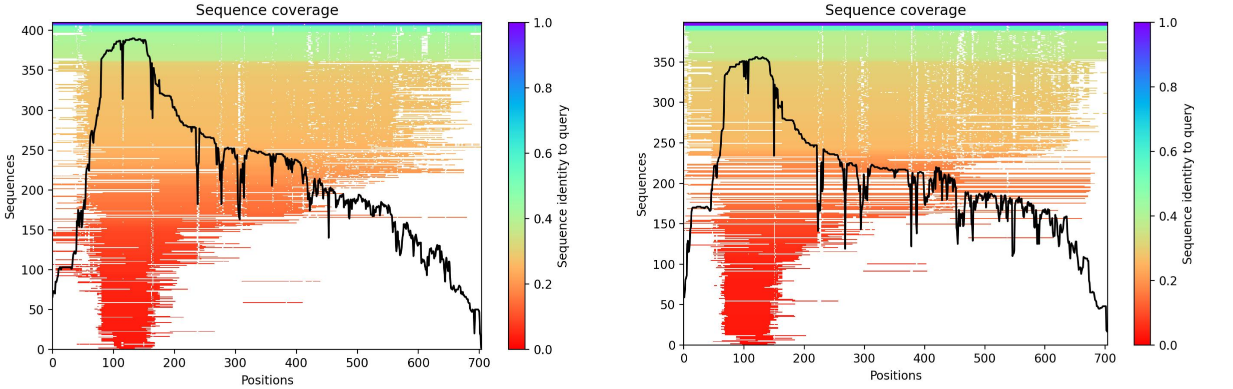

Supplementary Figure S7. Number of sequences at each position. ORF2 amino acid sequences for (A) GDYJ and (B) GDZJ. A higher number of sequence matches is associated with greater accuracy in the predictions.
