# Supplementary material for: Isolation, Characterization, and Comparative Analysis of Two Subtypes of Goose Astrovirus in Guangdong Province, China
Source: Microorganisms. 2025 Apr 30;13(5):1037. doi: 10.3390/microorganisms13051037 (PMC12114045; doi:10.3390/microorganisms13051037)
Supplement: Supplementary file 1 [file microorganisms-13-01037-s001.zip › microorganisms-3524754 Supplementary Table S1.pdf]

Supplementary Table S1. Mixed infections in 385 GAstV-positive samples with other viruses.

	single viral infections	sample size	proportion %	dual viral infections	sample size	proportion %	triple viral infections	sample size	proportion %
	GRV	10	2.6%	TMUV+GoCV	7	1.8%	TMUV+GRV+GoCV	3	0.8%
	GPV	9	2.3%	GRV+GPV	2	0.5%	TMUV+GPV+GoCV	2	0.5%
	FAdV	2	0.5%	GRV+GoCV	21	5.5%	GRV+GPV+GoCV	8	2.1%
	GoCV	174	45.2%	GPV+GoCV	49	12.7%	GRV+GPV+FAdV	1	0.3%
				FAdV+GoCV	15	3.9%	GRV+FAdV+GoCV	2	0.5%
							GPV+FAdV+GoCV	5	1.3%
**Total**		**195**	**50.7%**		**94**	**24.4%**		**21**	**5.5%**
